# Integrative single-cell analysis reveals immunogenic cell death–associated heterogeneity and identifies a robust prognostic signature in osteosarcoma with experimental validation

**DOI:** 10.3389/fphar.2026.1792174

**Published:** 2026-03-23

**Authors:** Zhaochen Xu, Jiangbo Han, Meng Zhang, Fei Chen, Hongli Deng, Weiguo Bian

**Affiliations:** 1 Department of Orthopedics, The First Affiliated Hospital of Xi’an JiaoTong University, Xi’an, China; 2 Xi'An Jiao Tong University, Xian Honghui Hospital, Department Emergency, Xi’an, China

**Keywords:** cell–cell communication, immunogenic cell death, osteosarcoma, prognostic model, single-cell RNA sequencing, tumor heterogeneity

## Abstract

**Background:**

Osteosarcoma is a highly aggressive primary malignant bone tumor characterized by pronounced intratumoral heterogeneity and an immunosuppressive tumor microenvironment. Immunogenic cell death (ICD), a regulated form of cell death capable of activating antitumor immunity, has been implicated in cancer progression and treatment response. However, the cell type–specific distribution of ICD features and their clinical relevance in osteosarcoma remain poorly defined.

**Methods:**

Single-cell RNA sequencing data, multiple bulk transcriptomic cohorts, and clinical follow-up information were integrated to characterize ICD-related transcriptional features in osteosarcoma. scRNA-seq analysis of 11 osteosarcoma samples from GSE152048 was used to define cellular composition and ICD activity at single-cell resolution. At the bulk level, the TCGA-TARGET cohort served as the training set, with GSE16091 and GSE21257 as validation cohorts. An ICD-related prognostic signature was constructed using WGCNA and ensemble machine learning. Pathway activity, cell–cell communication, and drug sensitivity were further analyzed. Functional assays in MG-63 and U-2OS cells were performed to validate the role of the key gene NAP1L1.

**Results:**

Single-cell analysis revealed pronounced cell type–dependent heterogeneity of ICD activity, with macrophages showing the highest ICD scores and epithelial-like tumor cells the lowest. The ICD-based prognostic model consistently stratified patient survival across all cohorts. High-risk tumors exhibited enrichment of inflammatory and stress-related pathways, including TNFA_SIGNALING_VIA_NFKB, COMPLEMENT, and COAGULATION, whereas low-risk tumors were associated with WNT_BETA_CATENIN, HEDGEHOG, and KRAS_SIGNALING_DN pathways. Cell–cell communication analysis demonstrated increased interaction frequency and signaling strength between tumor cells and immune or stromal cells in the high-risk group. Drug sensitivity prediction indicated lower IC50 values in high-risk patients for Camptothecin (p = 0.00031), Cytarabine (p = 0.013), Sorafenib (p = 0.000083), and SN-38 (p = 0.0042). Functional experiments showed that NAP1L1 overexpression promoted proliferation, migration, invasion, and clonogenicity in osteosarcoma cells.

**Conclusion:**

This study delineates ICD-related transcriptional heterogeneity in osteosarcoma at single-cell resolution and establishes a robust ICD-based prognostic signature. The findings suggest that ICD reflects an integrated tumor state shaped by cellular programs and microenvironmental interactions, providing preliminary evidence for its utility in risk stratification and therapeutic exploration, which warrants further prospective validation.

## 1 Introduction

Osteosarcoma is the most common primary malignant bone tumor (Damron et al., 2007). Adolescents and young adults account for approximately 60%–70% of all cases (Nie and Peng, 2018). In this population, osteosarcoma is associated with a high risk of disease-related mortality (Fritz et al., 2025). Clinical outcomes vary substantially among patients (Xin and Wei, 2020). Such variability cannot be adequately explained by tumor stage or histological features alone. Multidrug chemotherapy regimens based on methotrexate, doxorubicin, and cisplatin have become the standard treatment strategy for osteosarcoma (Zhang et al., 2020). This approach has improved the 5-year overall survival (OS) rate of patients with localized disease to approximately 60%–70% (Ottaviani and Jaffe, 2009; Papakonstantinou et al., 2024). However, in metastatic or recurrent osteosarcoma, the 5-year OS rate remains generally below 20%–30% (Longhi et al., 2006). No substantial improvement has been observed over the past few decades (Kleinerman, 2016). Extensive clinical observations show that even with similar treatment regimens, patients still show significant differentiation (Tirtei et al., 2021). These differences appear in disease progression speed, metastatic tendency, and treatment response. This suggests a highly complex and insufficiently characterized biological heterogeneity within osteosarcoma (Liu et al., 2024). Such heterogeneity is considered an important basis for therapeutic resistance and immune escape (Wang H. T. et al., 2025). It also constitutes a major bottleneck in the current development of precision treatment for osteosarcoma (Xu et al., 2025).

In tumor biology, modes of cell death have long been regarded as important regulators of tumor progression and treatment response (Peng et al., 2022). In recent years, increasing evidence has indicated that different forms of cell death do not merely determine whether tumor cells survive (Qi et al., 2022). They also play a critical role in shaping the tumor immune microenvironment (Hu et al., 2025). Among these, immunogenic cell death (ICD) is characterized by the exposure and release of specific molecular signals during cell death (Fucikova et al., 2020). These signals influence antigen presentation and immune cell recruitment (Tesniere et al., 2008). As a result, ICD has been increasingly recognized as a functional link between tumor intrinsic states and antitumor immune responses (Aaes and Vandenabeele, 2021). Previous studies across multiple solid tumors have reported that ICD related transcriptional or protein features are closely associated with immune infiltration patterns, responses to immunotherapy, and patient prognosis. However, most of these findings are derived from bulk level tumor analyses (Han X. et al., 2024). Direct evidence regarding how ICD states are distributed across different cell types, and whether substantial cell state–specific differences exist, remains limited (Im and Kim, 2023).

These issues are particularly pronounced in osteosarcoma. On one hand, osteosarcoma exhibits highly complex cellular composition (Asmar et al., 2025). Distinct malignant cell subpopulations, as well as immune and stromal cells, display marked differences in metabolic states, stress responses, and immunoregulatory capacity (Wang H. et al., 2025). On the other hand, most existing studies investigating cell death–related features in osteosarcoma rely on bulk transcriptomic data to construct risk models or molecular classifications (Hua et al., 2022). While statistically feasible, this analytical strategy makes it difficult to determine the cellular origin of observed signals (Suvà and Tirosh, 2019). It also limits the ability to capture dynamic cell states during tumor evolution (Ke et al., 2022). In addition, many previously reported prognostic models are based on a single algorithm for feature selection (Yao et al., 2024). Their robustness and generalizability across independent external cohorts therefore remain uncertain (Wu et al., 2023). Under these circumstances, discussing ICD or other cell death–related features solely at the bulk level is insufficient to explain the pronounced clinical heterogeneity observed in osteosarcoma. This limitation also restricts the potential application of such features in precision stratification and treatment decision-making (Tschodu et al., 2023).

Based on these considerations, the present study aims to re-evaluate the biological implications of ICD in osteosarcoma at both cellular resolution and system level, rather than treating ICD as a statistical summary of predefined genes. ICD related signals may arise from multiple malignant cell subpopulations and tumor microenvironment cells (Zhou et al., 2019). These signals may also undergo dynamic changes during tumor evolution. Relying solely on bulk transcriptomic measurements is therefore unlikely to accurately capture their true biological states (Quek et al., 2024). To address this issue, single-cell RNA sequencing data were integrated with multiple independent bulk transcriptomic cohorts and corresponding clinical follow-up information. This approach enabled the characterization of ICD distribution across cell types and cell states, as well as its association with the tumor microenvironment. On this basis, a robust ICD related risk model was constructed using co-expression network analysis combined with an integrated machine-learning framework, and applied to patient stratification and prognostic assessment. Furthermore, pathway characteristics, cell–cell communication networks, and drug sensitivity profiles were analyzed to systematically depict molecular ecosystem differences between distinct ICD risk states. Key genes were subsequently validated through *in vitro* functional experiments to ensure that the computational findings were supported by biological evidence. Through this multi-level analytical framework, this study seeks to provide more interpretable evidence for understanding ICD related heterogeneity and its potential clinical relevance in osteosarcoma.

## 2 Materials and methods

### 2.1 Data sources

In this study, bulk RNA sequencing data and corresponding clinical follow-up information used were primarily obtained from the TCGA-TARGET osteosarcoma cohort. This cohort was used for the construction and training of the ICD related prognostic model. The GSE16091 and GSE21257 cohorts were used as independent external validation datasets. All samples included in the analysis had complete gene expression matrices and OS information. Single-cell RNA sequencing (scRNA-seq) data were obtained from the GSE152048 dataset in the Gene Expression Omnibus (GEO) database. This dataset contained samples from 11 osteosarcoma patients and was used to characterize transcriptional features and ICD activity distribution across different cell types in the tumor microenvironment.

### 2.2 ICD related gene set construction

The ICD related gene set was constructed by integrating gene information associated with immunogenic cell death from the GeneCards database and published literature. To ensure the reliability of the gene set and minimize noise from weak text-mining associations, we selected candidate genes with a relevance score greater than five from the GeneCards database. Subsequently, reported core regulators of ICD were incorporated based on consensus reviews (Garg et al., 2015), resulting in a focused and robust signature. After manual curation and removal of redundant genes, the final ICD-related candidate gene set was used for subsequent single-cell ICD score calculation, co-expression network analysis, and prognostic model construction.

### 2.3 Data preprocessing

Bulk transcriptomic data were uniformly preprocessed prior to analysis. For expression data in raw count or transcripts per million (TPM) format, log_2_ (x + 1) transformation was applied to reduce the influence of extreme values. Only genes with stable expression within each dataset were retained for downstream analyses. Samples with missing key clinical follow-up information, including survival time or survival status, were excluded. Overall survival time was defined as the interval from initial diagnosis to death or last follow-up. Survival status was coded as death or censoring.

### 2.4 scRNA-seq analysis

scRNA-seq data were analyzed using the Seurat R package (Stuart et al., 2019). Seurat objects were first constructed, and the number of detected genes, unique molecular identifier (UMI) counts, and the proportion of mitochondrial genes were calculated for each cell. Quality control criteria were defined as follows. Cells with 200–7,000 detected genes, mitochondrial gene proportion below 10%, and erythrocyte-related gene proportion below 1% were retained. Potential doublets were identified and removed using the DoubletFinder algorithm. After normalization, principal component analysis (PCA) was performed for dimensionality reduction. Unsupervised clustering was conducted using the Louvain algorithm. Two-dimensional visualization was performed using t-distributed stochastic neighbor embedding (tSNE). Major cell types were annotated based on previously reported canonical marker genes. Based on the ICD related gene set, single-cell–level ICD activity scores were calculated using the AUCell method. These scores were mapped onto the tSNE space to visualize ICD state distributions across different cell types and subpopulations.

### 2.5 Identification of ICD related gene modules

Weighted gene co-expression network analysis (WGCNA) was performed on bulk transcriptomic data from the TCGA-TARGET osteosarcoma cohort to identify key gene modules associated with ICD. An appropriate soft-thresholding power was selected according to the scale-free topology criterion to construct a weighted co-expression network. Gene modules were identified based on the topological overlap matrix. Correlations between module eigengenes and ICD single-sample gene set enrichment analysis (ssGSEA) scores were calculated (Jin et al., 2021). Modules showing significant associations with ICD were selected for subsequent feature gene screening and prognostic model construction.

### 2.6 Construction of the prognostic signature

Based on the ICD related genes identified by WGCNA, multiple machine learning–based survival prediction models were constructed (Langfelder and Horvath, 2008). These included Random Survival Forest (RSF), least absolute shrinkage and selection operator (LASSO), Elastic Net, Ridge regression, and CoxBoost. All models were optimized using cross-validation. Time-dependent receiver operating characteristic (ROC) curve area under the curve (AUC) was used as the primary evaluation metric (Blanche and Blanche, 2019). By comparing predictive performance across the training cohort and external validation cohorts, the model with optimal overall performance and stability was selected. The RSF model was ultimately used to construct the ICD related risk score. Patients were stratified into high-risk and low-risk groups according to the median risk score.

### 2.7 Functional enrichment analysis

To characterize biological functional differences associated with ICD risk stratification, gene set enrichment analysis (GSEA) and gene set variation analysis (GSVA) were performed to compare high-risk and low-risk groups. GSEA was conducted using Hallmark gene sets from the Molecular Signatures Database (MSigDB) and implemented with the clusterProfiler R package. An adjusted p < 0.05 was considered statistically significant. GSVA was performed using the GSVA R package to calculate pathway activity scores for each sample. Differences in pathway activity between risk groups were assessed using the limma framework.

### 2.8 Cell–cell communication analysis

Cell–cell communication within the osteosarcoma tumor microenvironment was inferred based on single-cell transcriptomic data using the CellChat R package. Cell–cell communication networks were separately constructed for high-risk and low-risk groups. The number of interactions represented the inferred frequency of communication events between different cell types. Interaction strength represented the aggregated communication probability. Differences in communication patterns between high-risk and low-risk groups were further compared at the signaling pathway level to identify key pathways associated with ICD risk stratification.

### 2.9 Drug sensitivity prediction

To evaluate the association between ICD risk stratification and potential therapeutic response, drug sensitivity was predicted based on the Genomics of Drug Sensitivity in Cancer (GDSC) database using the pRRophetic R package. The half-maximal inhibitory concentration (IC50) for multiple anticancer drugs was estimated for each sample (Sebaugh, 2011). Differences in predicted IC50 values between high-risk and low-risk groups were assessed using the Wilcoxon rank-sum test. Drugs showing statistically significant differences were selected for result presentation.

### 2.10 *In vitro* functional validation of key gene

To validate the biological function of the key gene NAP1L1 identified in the ICD related risk model, *in vitro* functional experiments were conducted using the human osteosarcoma cell lines MG-63 and U-2OS. Both cell lines were obtained from the Cell Bank of the Chinese Academy of Sciences and cultured in Dulbecco’s modified Eagle’s medium supplemented with 10% fetal bovine serum and 1% penicillin–streptomycin (Pautke et al., 2004). Cells were maintained at 37 °C in a humidified incubator with 5% CO_2_. All experiments were performed using cells in the logarithmic growth phase.

For protein analysis, cells were washed with phosphate-buffered saline and lysed with RIPA buffer containing protease inhibitors. After centrifugation at 4 °C, supernatants were collected. Protein concentration was determined using the bicinchoninic acid assay. Equal amounts of protein were separated by SDS–PAGE and transferred onto polyvinylidene fluoride membranes. After blocking, membranes were incubated with primary antibodies against NAP1L1 and the internal control protein GAPDH, followed by incubation with horseradish peroxidase–conjugated secondary antibodies. Protein signals were visualized using chemiluminescence, and band intensities were quantified using ImageJ software.

An overexpression vector containing the full-length coding sequence of NAP1L1 was constructed, with an empty vector used as a negative control. Recombinant plasmids and packaging plasmids were co-transfected into HEK293T cells to generate lentiviral particles. Viral supernatants were collected at 48 h and 72 h after transfection and used to infect MG-63 and U-2OS cells in the presence of polybrene. Puromycin selection was applied 48 h after infection to establish stable overexpression cell lines. NAP1L1 protein expression was confirmed by Western blot analysis.

Cell proliferation was assessed using the Cell Counting Kit-8 (CCK-8) assay. Stably transfected cells were seeded into 96-well plates at a density of 2 × 10^3^ cells per well. CCK-8 reagent was added at 0, 24, 48, and 72 h. Absorbance at 450 nm was measured to evaluate cell growth over time. Long-term proliferative capacity was assessed using colony formation assays. Stably transfected cells were seeded into six-well plates at a density of 500 cells per well and cultured for 10–14 days. After visible colonies formed, cells were fixed with 4% paraformaldehyde and stained with crystal violet. Colonies containing more than 50 cells were counted using ImageJ software (Grishagin, 2015).

Cell migration ability was evaluated using wound healing assays. Stably transfected cells were seeded into six-well plates. When cell confluence reached approximately 90%, a scratch was created in the cell monolayer using a sterile pipette tip. Detached cells were removed, and serum-free medium was added. Images were captured at 0 h and 24 h. Relative migration rates were calculated based on changes in wound width.

Cell migration and invasion were further assessed using Transwell assays. For migration assays, serum-starved cells were seeded into the upper chamber, and medium containing a high concentration of fetal bovine serum was added to the lower chamber as a chemoattractant. After incubation, migrated cells were fixed, stained, and counted. For invasion assays, the upper chamber was pre-coated with Matrigel to simulate the extracellular matrix environment. The remaining procedures were identical to those used for migration assays. Cells were counted in multiple randomly selected fields for each sample. All *in vitro* experiments were independently repeated at least three times.

### 2.11 Statistical analysis

All statistical analyses were performed using R software (v4.5.1). Comparisons between groups for continuous variables were conducted using the Wilcoxon rank-sum test. Correlation analyses were performed using Spearman’s correlation coefficient. Survival analyses were conducted using the Kaplan-Meier method and log-rank test. All statistical tests were two-sided, and p < 0.05 was considered statistically significant.

## 3 Results

**FIGURE 1:**
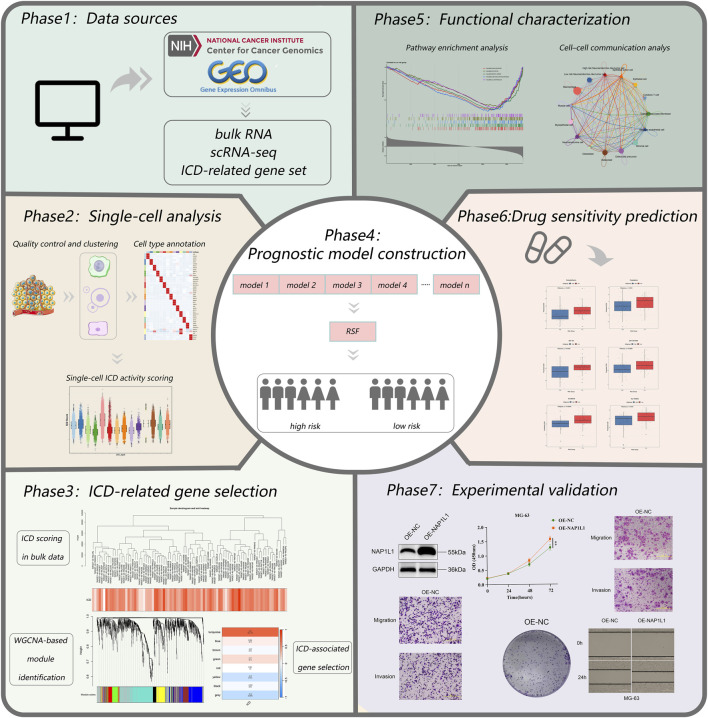
Study flowchart.

All analytical processes are illustrated in the flowchart (Figure 1).

### 3.1 Cellular composition of the osteosarcoma tumor microenvironment and distribution of ICD features at single-cell resolution

**FIGURE 2:**
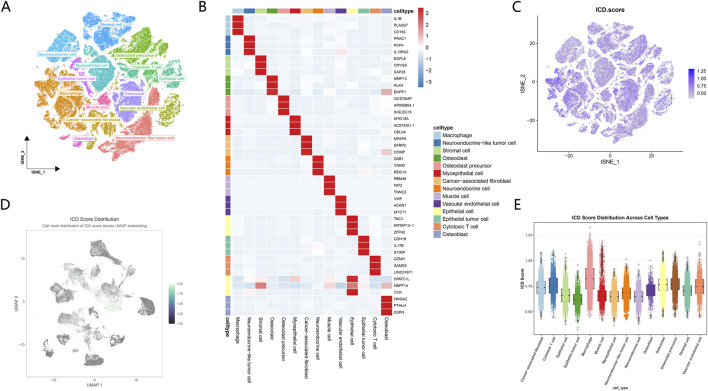
Single-cell landscape of the osteosarcoma tumor microenvironment and distribution of ICD features. **(A)** tSNE projection of single cells from osteosarcoma samples, colored by annotated cell types, including tumor cells, immune cells, and stromal-related populations. Each point represents 1 cell. **(B)** Heatmap showing the expression patterns of representative marker genes across different cell types. Rows indicate marker genes and columns represent cell types. Color scale denotes scaled gene expression levels. **(C)** tSNE embedding colored by ICD score at the single-cell level, illustrating the spatial distribution of ICD related transcriptional activity across the tumor microenvironment. **(D)** UMAP projection displaying the continuous distribution of ICD score among individual cells. Color gradient indicates relative ICD score intensity. **(E)** Boxplot showing the distribution of ICD scores across different cell types. Each dot represents an individual cell. Center line indicates the median, and boxes denote the interquartile range.

After dimensionality reduction and clustering of osteosarcoma tumor samples at single-cell resolution, cells formed multiple well-defined clusters in low-dimensional space. Based on transcriptional characteristics, these cells were classified into malignant cells, immune cells, and multiple stromal-related populations. Identified cell types included epithelial tumor cells, neuroendocrine-like tumor cells, macrophages, cytotoxic T cells, osteoblasts, osteoclasts and their precursors, cancer-associated fibroblasts, vascular endothelial cells, stromal cells, and muscle-like cells. Each cell population exhibited a relatively compact distribution in the tSNE space, with clear boundaries between clusters. No obvious continuous transitions or cluster mixing were observed (Figure 2A).

At the level of marker gene expression, distinct cell populations displayed highly specific transcriptional profiles. Macrophages showed markedly elevated expression of IL1B, PLA2G7, and CD163, while these genes were maintained at low levels in other cell types. Among bone metabolism–related cells, osteoclasts were characterized by high expression of MMP13, KLK4, and ENPP1, whereas osteoclast precursors exhibited prominent expression of OCSTAMP, AP000904.1, and SIGLEC15. In vascular-associated cells, vascular endothelial cells showed strong expression of VWF, ACKR1, and MYCT1. Among tumor-related populations, epithelial tumor cells and neuroendocrine-like tumor cells displayed clearly distinguishable expression patterns, which were distinct from those of immune and stromal cells (Figure 2B).

Based on these annotations, ICD scores were calculated at the single-cell level. After projection of ICD scores onto the tSNE space, a pronounced spatial heterogeneity was observed. High ICD scores were not uniformly distributed across all cell populations. Instead, they showed localized enrichment within specific clusters. Substantial differences in ICD scores were observed among cells located in adjacent regions, indicating that ICD related transcriptional features were not globally activated across the tumor microenvironment (Figure 2C).

A similar heterogeneous pattern was observed when ICD scores were visualized in the UMAP space. Certain cellular regions were dominated by higher ICD scores, whereas other regions remained at lower levels. Even within the same cell population, ICD scores exhibited gradient changes and discrete distributions, reflecting differences at the cellular state level (Figure 2D).

Comparison of ICD scores across different cell types revealed clear distributional differences among cell populations. Macrophages exhibited the highest ICD scores, with both median values and overall distributions markedly exceeding those of other cell types. In contrast, epithelial tumor cells showed the lowest ICD scores, with distributions largely concentrated in the low-value range. ICD scores in other cell types generally fell between these two extremes, and the distribution widths varied across populations. Notably, ICD scores remained dispersed even within the same cell type, indicating that ICD related transcriptional features were not uniformly present at the cellular level (Figure 2E). Together, these results demonstrate that ICD related transcriptional features in the osteosarcoma tumor microenvironment exhibit significant heterogeneity both between and within cell types. Their distribution follows a clear spatial structure rather than random fluctuation.

### 3.2 Identification of ICD related co-expression modules and their associated features

**FIGURE 3:**
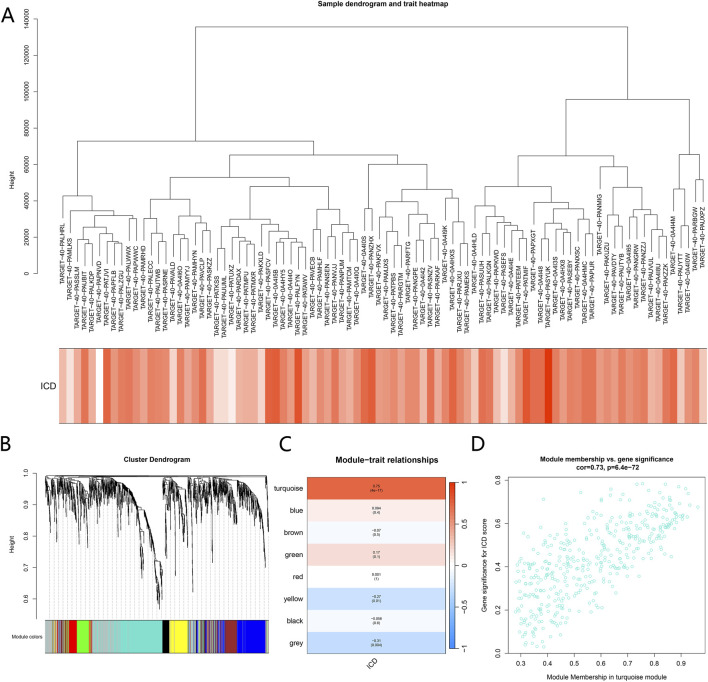
Identification of immunogenic cell death (ICD) related gene co-expression modules using WGCNA. **(A)** Sample dendrogram based on hierarchical clustering of gene expression profiles, with the ICD trait shown as a sample annotation heatmap below, illustrating the distribution of ICD status across samples. **(B)** Hierarchical clustering dendrogram of genes constructed by WGCNA. Different colors represent distinct gene co-expression modules identified by dynamic tree cutting. **(C)** Heatmap showing correlations between module eigengenes and the ICD trait. Colors indicate the direction and strength of correlations. Numbers represent correlation coefficients, with corresponding P values in parentheses. **(D)** Scatter plot illustrating the relationship between module membership and gene significance for ICD within the turquoise module. Each dot represents a gene, showing the association between intramodular connectivity and ICD relevance.

To further identify gene co-expression structures associated with the ICD phenotype at the transcriptome-wide level, weighted gene co-expression networks were constructed based on gene expression data from osteosarcoma samples. Sample-level hierarchical clustering showed no obvious outliers across samples. ICD phenotypes exhibited a continuous distribution among samples and did not display abrupt changes along clustering branches (Figure 3A).

At the gene level, multiple co-expression modules were identified through hierarchical clustering combined with the dynamic tree-cut algorithm. These modules differed markedly in gene number and internal structure. In the clustering dendrogram, individual modules formed relatively independent branch structures, indicating stable module assignment (Figure 3B).

Correlation analysis between module eigengenes and the ICD phenotype revealed substantial differences in the strength of association across modules. Among them, the turquoise module showed the strongest positive correlation with the ICD phenotype, with a correlation coefficient of 0.75 (p < 0.0001). In contrast, other modules exhibited weaker associations with ICD, with some showing negative correlations or correlations close to zero (Figure 3C).

Based on these results, the turquoise module was selected as the core ICD related module for further analysis. Within this module, module membership was strongly positively correlated with ICD gene significance (cor = 0.73, p = 6.4e−72). The scatter plot showed that most genes simultaneously exhibited high module membership and high ICD significance. This distribution indicates that the centrality of key genes within the co-expression network is consistent with the strength of their association with the ICD phenotype (Figure 3D).

### 3.3 Construction of the ICD related prognostic model and validation across multiple cohorts

**FIGURE 4:**
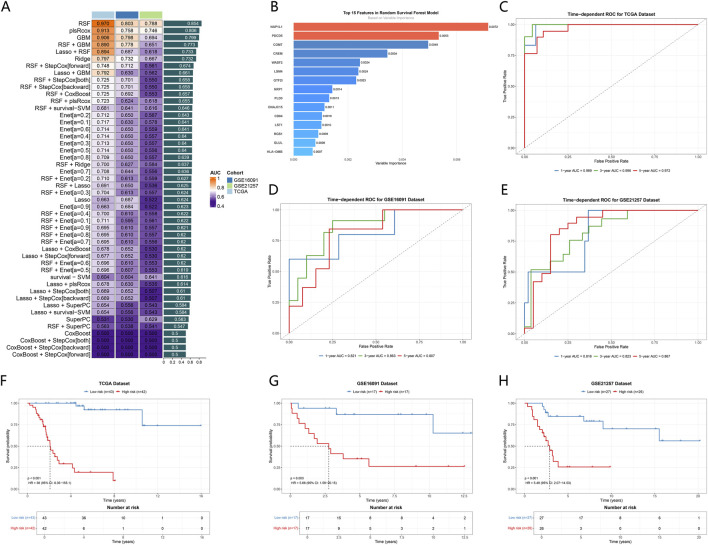
Construction and validation of an ICD related prognostic model across multiple cohorts. **(A)** Heatmap summarizing the area AUC values of different survival models across the GSE16091, GSE21257, and TCGA cohorts. Rows represent modeling strategies, and columns represent datasets. **(B)** Top 15 features ranked by variable importance in the RSF model. Bar length indicates relative importance of each gene. **(C)** Time-dependent ROC curves for 1-year, 3-year, and 5-year overall survival prediction in the TCGA dataset. **(D)** Time-dependent ROC curves for 1-year, 3-year, and 5-year overall survival prediction in the GSE16091 dataset. **(E)** Time-dependent ROC curves for 1-year, 3-year, and 5-year overall survival prediction in the GSE21257 dataset. **(F)** Kaplan–Meier survival curves comparing overall survival between high risk and low risk groups in the TCGA dataset. **(G)** Kaplan–Meier survival curves comparing overall survival between high-risk and low-risk groups in the GSE16091 dataset. **(H)** Kaplan–Meier survival curves comparing overall survival between high-risk and low-risk groups in the GSE21257 dataset.

After identifying the ICD-related co-expression modules, we constructed multiple survival prediction models based on the candidate genes and systematically compared their performance across different datasets. While the predictive ability of different algorithms varied among the three cohorts, the Random Survival Forest (RSF) model consistently exhibited the highest stability and AUC values in the GSE16091, GSE21257, and TCGA cohorts. Notably, its overall performance was superior to that of conventional linear models (such as Cox regression and LASSO) and other integrated approaches (Figure 4A), likely due to RSF’s inherent ability to capture complex, non-linear interactions within high-dimensional transcriptomic data. Consequently, RSF was selected to construct the final ICD-related prognostic signature.

Within the RSF model, feature genes with the highest contributions were identified according to variable importance ranking. The results showed that NAP1L1, PDCD5, COMT, and CREM occupied higher weights in the model. The importance values of different genes displayed a graded distribution rather than a sharp cutoff (Figure 4B).

Based on the RSF model, time-dependent predictive performance was further evaluated across different cohorts. In the TCGA cohort, the AUC values at 1, 3, and 5 years were 0.989, 0.996, and 0.972, respectively, indicating high predictive accuracy (Figure 4C). In the GSE16091 cohort, the corresponding AUC values were 0.821, 0.863, and 0.807 (Figure 4D). In the GSE21257 cohort, the AUC values at 1, 3, and 5 years were 0.816, 0.823, and 0.867. These results indicate that the model maintained good robustness and reproducibility across independent cohorts (Figure 4E).

After patients were stratified into high-risk and low-risk groups based on RSF-derived risk scores, consistent survival differences were observed across the TCGA, GSE16091, and GSE21257 cohorts. Overall survival was lower in the high-risk group than in the low-risk group in all three datasets (Figures 4F–H).

### 3.4 Pathway characteristics associated with ICD risk stratification and their prognostic relevance

**FIGURE 5:**
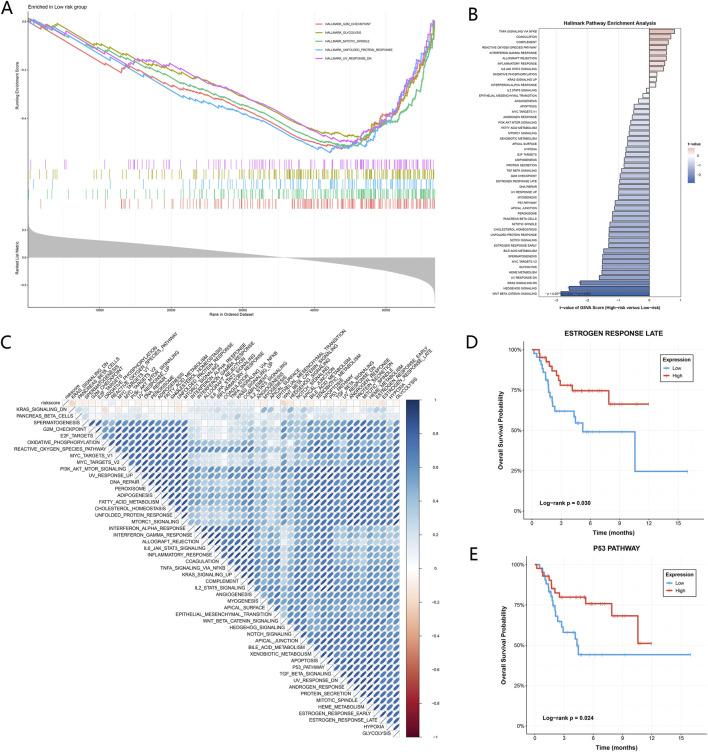
Pathway characteristics associated with ICD risk stratification and survival outcomes. **(A)** GSEA showing representative Hallmark pathways enriched in the low-risk group. Enrichment plots display running enrichment scores across the ranked gene list. **(B)** Hallmark pathway enrichment analysis based on GSVA, comparing pathway activity between high-risk and low-risk groups. Bars represent t-values of GSVA scores (high-risk *versus* low-risk). **(C)** Correlation matrix showing associations between ICD risk score and Hallmark pathway activities. Color intensity indicates the direction and strength of correlation. **(D)** Kaplan–Meier survival curves stratified by high and low activity of the ESTROGEN_RESPONSE_LATE pathway. **(E)** Kaplan–Meier survival curves stratified by high and low activity of the P53_PATHWAY.

Based on ICD risk stratification derived from the RSF model, pathway differences between high-risk and low-risk groups were analyzed. GSEA results showed that multiple Hallmark pathways were significantly enriched in the low-risk group. These pathways included G2M_CHECKPOINT, GLYCOLYSIS, MITOTIC_SPINDLE, UNFOLDED_PROTEIN_RESPONSE, and UV_RESPONSE_DN. Their enrichment curves exhibited consistent shifts along the ranked gene list (Figure 5A).

To systematically compare overall functional differences between the two groups, GSVA was further applied to quantify Hallmark pathway activity. The results showed that several inflammatory and immune-related pathways had higher scores in the high-risk group, mainly including TNFA_SIGNALING_VIA_NFKB, COAGULATION, and COMPLEMENT. In contrast, WNT_BETA_CATENIN_SIGNALING, HEDGEHOG_SIGNALING, and KRAS_SIGNALING_DN displayed relatively higher activity levels in the low-risk group (Figure 5B).

Correlation analysis between ICD risk scores and Hallmark pathway activity revealed widespread associations across multiple pathways. The direction and magnitude of correlations varied substantially among pathways. Overall, the correlations exhibited a modular distribution pattern rather than being dominated by a single pathway (Figure 5C).

Among these pathways, ESTROGEN_RESPONSE_LATE and P53_PATHWAY, which showed more pronounced associations with prognosis, were selected for further analysis. After patients were stratified according to pathway activity levels, both pathways demonstrated clear survival differences. Patients with higher ESTROGEN_RESPONSE_LATE activity exhibited significantly better overall survival than those with lower activity levels (Figure 5D). Similarly, higher activity of the P53_PATHWAY was associated with longer survival time (Figure 5E).

### 3.5 Cell-of-origin differences and molecular features associated with ICD risk stratification

**FIGURE 6:**
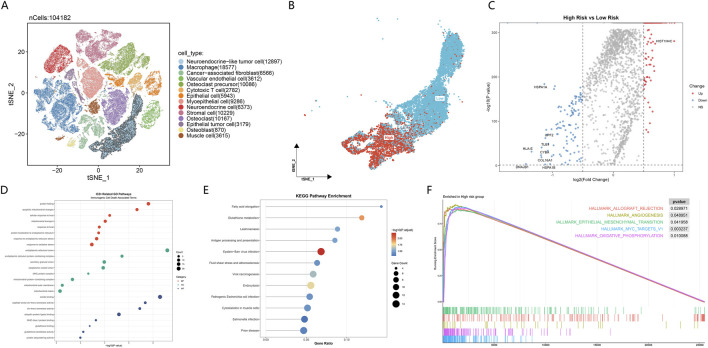
Single-cell distribution and functional characteristics associated with ICD risk stratification. **(A)** tSNE projection of single cells from osteosarcoma samples, colored by annotated cell types. Each dot represents 1 cell. **(B)** tSNE embedding showing the spatial distribution of ICD risk stratification at the single-cell level. Cells are colored according to high-risk and low-risk groups. **(C)** Volcano plot displaying differentially expressed genes between high-risk and low-risk groups. Red and blue dots indicate upregulated genes in high-risk and low-risk groups, respectively. **(D)** GO enrichment analysis of differentially expressed genes, including BP, CC, and MF categories. Bubble size represents gene counts and color indicates statistical significance. **(E)** KEGG pathway enrichment analysis of differentially expressed genes. Bubble size denotes the number of enriched genes and color represents adjusted P values. **(F)** GSEA showing representative Hallmark pathways enriched in the high-risk group. Enrichment plots display running enrichment scores across the ranked gene list.

At the single-cell level, the overall cellular composition of the osteosarcoma tumor microenvironment was visualized. A total of 104,182 cells were included, encompassing tumor cells, immune cells, and multiple stromal-related cell populations. Each cell population formed a stable distribution pattern in the tSNE space (Figure 6A).

After mapping ICD risk stratification onto the single-cell embedding space, a clear spatial separation between high-risk and low-risk cells was observed. Cells associated with the high-risk group were mainly concentrated in the lower region of the tSNE plot, whereas low-risk–associated cells were more frequently distributed in the upper region. The overlap between the two groups in low-dimensional space was relatively limited (Figure 6B).

Differential expression analysis between the high-risk and low-risk groups revealed multiple representative genes showing consistent directional changes. The volcano plot demonstrated that genes such as HIST1H4C were significantly upregulated in the high-risk group, whereas HSPA1A, DNAJB1, and HLA-E were relatively more highly expressed in the low-risk group (Figure 6C). These genes showed clear separation in both effect size and statistical significance, indicating distinct transcriptional differences between the two risk groups.

Functional annotation of differentially expressed genes revealed that distinct Gene Ontology (GO) categories were associated with specific biological tasks. At the level of Biological Process (BP), enriched terms were mainly related to protein folding, response to oxidative stress, and response to endoplasmic reticulum stress. At the level of Cellular Component (CC), associated genes were primarily localized to the endoplasmic reticulum lumen, secretory granule lumen, cytoplasmic vesicle lumen, and mitochondrial matrix. At the level of Molecular Function (MF), enriched terms were concentrated in amide binding, peptidyl-prolyl cis-trans isomerase activity, cis-trans isomerase activity, and ubiquitin protein ligase binding (Figure 6D).

At the level of Kyoto Encyclopedia of Genes and Genomes (KEGG) pathways, differentially expressed genes were significantly enriched in multiple pathways with clear functional relevance. Metabolic and redox-related pathways included Glutathione metabolism and Fatty acid elongation. Immune- and antigen-processing–related pathways mainly involved Antigen processing and presentation. In addition, differentially expressed genes were enriched in several disease- and stress-related pathways, including Epstein–Barr virus infection, Viral carcinogenesis, Leishmaniasis, *Salmonella* infection, Pathogenic *Escherichia coli* infection, and Prion disease. Among pathways related to cellular processes, Endocytosis and Cytoskeleton in muscle cells also showed significant enrichment (Figure 6E).

Further GSEA of pathway-level global trends revealed that multiple Hallmark pathways were significantly enriched in the high-risk group. These included HALLMARK_ALLOGRAFT_REJECTION, HALLMARK_ANGIOGENESIS, HALLMARK_EPITHELIAL_MESENCHYMAL_TRANSITION, HALLMARK_MYC_TARGETS_V1, and HALLMARK_OXIDATIVE_PHOSPHORYLATION. Their enrichment curves showed consistent shifts along the ranked gene list (Figure 6F).

### 3.6 Cell–cell communication features associated with ICD risk stratification

**FIGURE 7:**
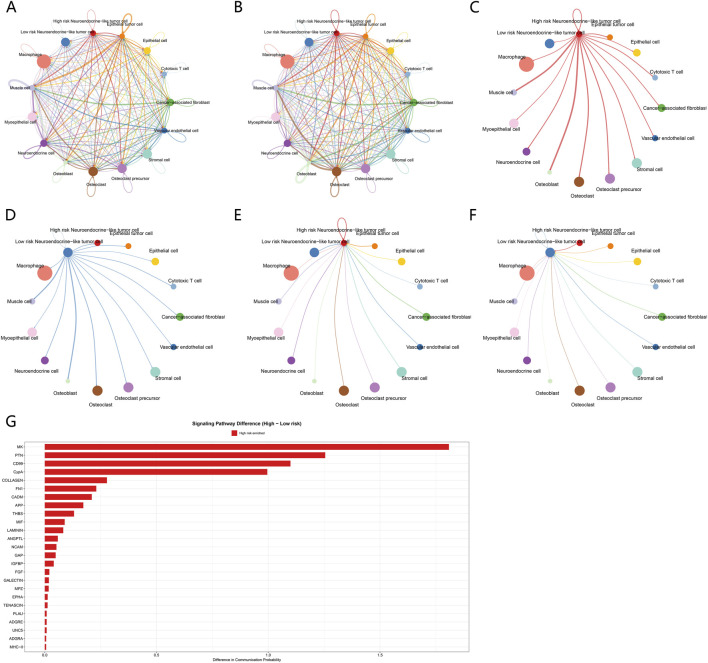
Cell–cell communication patterns associated with ICD risk stratification. **(A)** Global cell–cell communication network showing the number of interactions among major cell types in the osteosarcoma tumor microenvironment. Node size represents the total number of inferred ligand–receptor interaction events involving each cell type, reflecting the frequency of cell–cell communication. **(B)** Comparison of global cell–cell communication networks between high-risk and low-risk groups based on interaction strength, representing the aggregated signaling intensity of inferred cell–cell communications. Edge width indicates the overall communication strength between cell types. **(C)** Cell–cell communication network centered on high-risk neuroendocrine-like tumor cells, illustrating outgoing signaling pathways based on communication weights, with edges representing pathway-weighted signals transmitted from tumor cells to other cell types. **(D)** Cell–cell communication network centered on low-risk neuroendocrine-like tumor cells, illustrating corresponding outgoing signaling pathways based on communication weights in the low-risk group. **(E)** Differential cell–cell communication network highlighting incoming signaling pathways to high-risk neuroendocrine-like tumor cells, with edge width indicating relative communication weight received from other cell types. **(F)** Differential cell–cell communication network highlighting incoming signaling pathways to low-risk neuroendocrine-like tumor cells, with edge width indicating relative communication weight received from other cell types. **(G)** Bar plot showing differences in pathway-level communication probability between high-risk and low-risk groups across representative signaling pathways. Positive values indicate higher communication probability in the high-risk group.

To further characterize cell–cell communication patterns within the tumor microenvironment under different ICD risk states, a systematic analysis of cell–cell communication was performed for the high-risk and low-risk groups. First, communication frequency between different cell types was evaluated based on the number of interactions (Figure 7A). The results showed that, compared with the low-risk group, the high-risk group exhibited a higher number of communication events between tumor cells and multiple immune and stromal cell types. This finding indicates that high-risk tumors are involved in more frequent intercellular information exchange at the global level. Subsequently, interaction strength was compared to reflect the intensity of signal transmission among established communication events (Figure 7B). The analysis demonstrated that the high-risk group showed not only increased communication frequency but also significantly enhanced overall communication strength. These results suggest that under high ICD risk conditions, signal transmission within the tumor microenvironment is synchronously amplified at both the frequency and intensity levels.

On this basis, pathway-level dissection of intercellular signaling was further conducted using communication weight, rather than relying solely on communication counts, to more accurately assess the relative contribution of different signaling pathways. Pathway-level analyses were performed from both outgoing and incoming signaling perspectives. In the analysis of outgoing signaling pathways (Figures 7C,D), tumor cells in the high-risk group, particularly neuroendocrine-like tumor cells, exhibited higher communication weights across multiple pathways. These cells transmitted stronger signals to surrounding immune and stromal cells. In contrast, outgoing signaling weights of the corresponding pathways were generally lower in the low-risk group. Consistently, in the analysis of incoming signaling pathways (Figures 7E,F), tumor cells in the high-risk group received higher communication weights from multiple pathways within the tumor microenvironment than those in the low-risk group. This finding indicates that high-risk tumor cells act as both more active signal senders and signal receivers within the cell–cell communication network.

Finally, direct comparison of pathway-level communication weights between the high-risk and low-risk groups identified a set of cell–cell communication pathways that were specifically enhanced in the high-risk state (Figure 7G). Among these, the MK, PTN, CD99, and CypA signaling pathways exhibited higher communication probabilities in the high-risk group. The enhancement of these pathways does not simply reflect an overall increase in communication frequency. Instead, it suggests selective remodeling of specific cell–cell communication axes within the tumor microenvironment under high ICD risk conditions.

### 3.7 Drug sensitivity differences associated with ICD risk stratification

Based on ICD risk scores, predicted drug sensitivity was compared between the high-risk and low-risk groups across multiple anticancer agents. The results showed significant differences in predicted half-maximal IC50 values between the two risk groups for several drugs.

**FIGURE 8:**
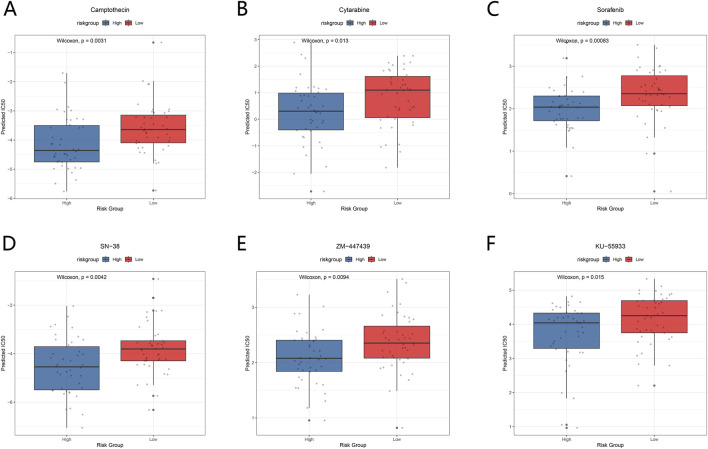
Predicted drug sensitivity differences between high-risk and low-risk groups based on ICD risk stratification. **(A)** Comparison of predicted IC50 values for Camptothecin between high-risk and low-risk groups. **(B)** Comparison of predicted IC50 values for Cytarabine between high-risk and low-risk groups. **(C)** Comparison of predicted IC50 values for Sorafenib between high-risk and low-risk groups. **(D)** Comparison of predicted IC50 values for SN-38 between high-risk and low-risk groups. **(E)** Comparison of predicted IC50 values for ZM-447439 between high-risk and low-risk groups. **(F)** Comparison of predicted IC50 values for KU-55933 between high-risk and low-risk groups.

Under Camptothecin treatment, the predicted IC50 was significantly lower in the high-risk group than in the low-risk group (Wilcoxon test, p < 0.001), indicating higher sensitivity to this drug in the high-risk group (Figure 8A). A similar trend was observed for Cytarabine, with the predicted IC50 being markedly lower in the high-risk group than in the low-risk group (p = 0.013) (Figure 8B). Under Sorafenib treatment, the high-risk group also exhibited a lower predicted IC50 compared with the low-risk group (p < 0.001) (Figure 8C).

In addition, for SN-38 and ZM-447439, the predicted IC50 values were significantly lower in the high-risk group than in the low-risk group, with statistically significant differences observed for both drugs (SN-38: p < 0.001; ZM-447439: p < 0.001) (Figures 8D,E). For KU-55933, the predicted IC50 in the high-risk group was also lower than that in the low-risk group (p = 0.015) (Figure 8F).

### 3.8 *In vitro* functional validation of NAP1L1 in osteosarcoma cells

**FIGURE 9:**
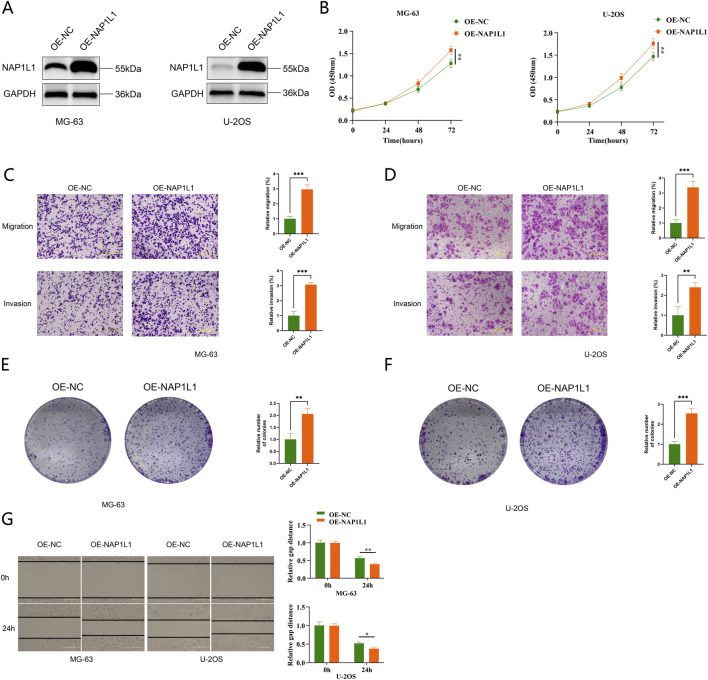
*In vitro* functional validation of NAP1L1 in osteosarcoma cell lines. **(A)** Western blot analysis showing NAP1L1 protein expression in MG-63 and U-2OS cells transfected with OE-NAP1L1 or negative control (OE-NC). GAPDH was used as a loading control. **(B)** Cell proliferation curves assessed by CCK-8 assay in MG-63 and U-2OS cells following NAP1L1 overexpression. OD values were measured at the indicated time points. **(C)** Representative images and quantification of Transwell migration and invasion assays in MG-63 cells transfected with OE-NAP1L1 or OE-NC. **(D)** Representative images and quantification of Transwell migration and invasion assays in U-2OS cells transfected with OE-NAP1L1 or OE-NC. **(E)** Colony formation assay and quantification in MG-63 cells after NAP1L1 overexpression. **(F)** Colony formation assay and quantification in U-2OS cells after NAP1L1 overexpression. **(G)** Wound-healing assay showing representative images and quantitative analysis of relative gap distance at 0 h and 24 h in MG-63 and U-2OS cells transfected with OE-NAP1L1 or OE-NC. *p < 0.05, **p < 0.01, ***p < 0.001.

Western blot analysis confirmed successful NAP1L1 overexpression in MG-63 and U-2OS cells, showing elevated protein levels in the OE-NAP1L1 group relative to controls (OE-NC) (Figure 9A).

Cell proliferation assays showed that NAP1L1 overexpression significantly enhanced proliferative capacity in both MG-63 and U-2OS cells. In the CCK-8 assay, the OD450 values of the OE-NAP1L1 group at 72 h were higher than those of the control group in both cell lines. The differences reached statistical significance in MG-63 and U-2OS cells (both p < 0.05) (Figure 9B).

Transwell assays demonstrated that, in MG-63 cells, the numbers of migrated and invaded cells were higher in the OE-NAP1L1 group than in the control group. Both differences showed high statistical significance (both p < 0.001) (Figure 9C). In U-2OS cells, NAP1L1 overexpression similarly promoted cell migration and invasion. The differences reached p < 0.001 for migration and p < 0.01 for invasion, respectively (Figure 9D).

Colony formation assays showed that, compared with the control group, the OE-NAP1L1 group formed a significantly greater number of colonies in MG-63 cells (p < 0.01) (Figure 9E). Similar results were observed in U-2OS cells, with a statistically significant increase in colony numbers (p < 0.001) (Figure 9F).

In addition, wound healing assays were used to dynamically assess cell migration. At 24 h, the relative wound distance in the OE-NAP1L1 group was significantly shorter than that in the control group in MG-63 cells (p < 0.01). A similar significant difference was also observed in U-2OS cells (p < 0.05) (Figure 9G).

## 4 Discussion

This study systematically characterized the pronounced heterogeneity of ICD related features in osteosarcoma at single-cell resolution. Single-cell analyses demonstrated that ICD scores exhibited marked non-uniform distributions in both malignant cells and tumor microenvironment cells. These differences could not be simply attributed to cell lineage alone. Within malignant cells, distinct tumor subpopulations showed clearly differentiated ICD states, with neuroendocrine-like tumor cells and epithelial tumor cells displaying substantially different ICD levels. In contrast to previous studies that primarily relied on bulk transcriptomic data and treated ICD as a tumor-wide property, the present study directly reveals at the single-cell level that ICD more closely reflects a dynamic cellular state rather than a fixed and stable phenotype (Liu et al., 2022; Han S. et al., 2024; Yang et al., 2022). This finding provides a cell-level explanation for the highly inconsistent ICD related signals observed in clinical samples. Notably, our single-cell analysis identified that macrophages exhibited the highest ICD scores among all cell types in the TME. We postulate that this observation reflects the functional “reactive state” of macrophages rather than their own cell death. As primary phagocytes, macrophages are recruited to engulf dying tumor cells (efferocytosis) and sense Damage-Associated Molecular Patterns (DAMPs) released during immunogenic cell death (Ge et al., 2022). This process triggers downstream signaling pathways, such as NF-κB and type I interferon responses, leading to the transcriptional upregulation of immune activation markers (e.g., CALR, HSP90AA1, CD80) that overlap significantly with the ICD gene signature. Thus, the elevated ICD scores in macrophages likely serve as a transcriptomic footprint of their active engagement in sensing and processing immunogenic signals from dying osteosarcoma cells, highlighting the extensive crosstalk between tumor cell death and innate immune activation.

Beyond tumor-intrinsic differences, this study further identified a systematic coupling between ICD risk stratification and the immune microenvironment in osteosarcoma. At the pathway level, the high-risk group was consistently enriched for inflammatory and immune-related pathways, including TNFA_SIGNALING_VIA_NFKB, COMPLEMENT, and COAGULATION. In contrast, the low-risk group showed relative enrichment of pathways associated with signaling suppression and differentiation, such as WNT_BETA_CATENIN_SIGNALING, HEDGEHOG_SIGNALING, and KRAS_SIGNALING_DN. Previous studies in osteosarcoma and other sarcomas have reported that the TNF–NFκB axis and complement coagulation cascades are closely associated with chronic inflammatory states, dysregulated immune cell recruitment, and poor prognosis (Shi et al., 2025; Lin et al., 2021). By comparison, WNT and Hedgehog signaling pathways are more frequently involved in the maintenance of tumor differentiation and immune exclusion (Luke et al., 2019; Li et al., 2019). The concordant directional changes of these pathways with ICD risk stratification observed in this study suggest that high ICD risk does not represent an isolated cell death signal. Instead, it reflects an integrated transcriptional state embedded within inflammatory and immune regulatory networks. Single-cell–level cell–cell communication analyses further support this interpretation. Under high ICD risk conditions, communication networks centered on neuroendocrine-like tumor cells were markedly enhanced in both communication frequency and interaction strength. These tumor cells formed high-intensity connections with macrophages, cancer-associated fibroblasts, and vascular endothelial cells, indicating global amplification of tumor–microenvironment signaling exchange.

In prognostic and treatment-related analyses, ICD risk stratification not only demonstrated stable survival predictive capacity but also delineated distinct signal dependency patterns. ICD risk scores constructed using the random survival forest model consistently separated patient subgroups with significantly different outcomes across multiple independent cohorts. Pathway analyses showed that high ICD risk states were closely associated with coordinated activation of programs such as MYC_TARGETS, OXIDATIVE_PHOSPHORYLATION, and EPITHELIAL_MESENCHYMAL_TRANSITION. Previous studies have suggested that ICD related molecular events are often accompanied by metabolic reprogramming, enhanced mitochondrial function, and the emergence of EMT phenotypes (Galluzzi et al., 2017; Liesenborghs et al., 2020). These changes increase tumor cell dependence on cell cycle progression and energy supply (Ekmekcioglu et al., 2016). Consistent with these observations, drug sensitivity prediction based on the Genomics of Drug Sensitivity in Cancer database revealed that the high ICD risk group exhibited significantly lower predicted IC50 values for multiple agents, including Camptothecin, Cytarabine, and notably Sorafenib (Yang et al., 2013). Although Camptothecin and Cytarabine are not standard-of-care for osteosarcoma, their identification reflects the high proliferative index and replicative stress characteristic of the high-risk group. Of greater clinical translational significance, the prediction of Sorafenib aligns compellingly with clinical evidence. Specifically, Grignani et al. demonstrated the therapeutic activity of Sorafenib in a non-randomised Phase two clinical trial (registered with ClinicalTrials.gov, number NCT01804374) for patients with unresectable high-grade osteosarcoma progressing after standard treatment (Grignani et al., 2015). Furthermore, Pignochino et al. provided preclinical validation showing that Sorafenib blocks tumor growth and metastasis *via* the ERK1/2 and MCL-1 pathways (Pignochino et al., 2009). This concordance suggests that our ICD-risk signature may capture specific tumor vulnerabilities, offering a potential tool to stratify patients who are most likely to benefit from targeted therapies like Sorafenib.

At a higher level, the ICD risk features identified in this study do not reflect the activity of a single signaling pathway. Instead, they represent the combined effects of multiple stress, inflammatory, and metabolic programs. By integrating single-cell transcriptomic data, bulk datasets, pathway analyses, and cell–cell communication networks, this study demonstrates that high ICD risk states are often associated with a composite phenotype characterized by high stress, high communication activity, and high invasiveness. This phenotype is particularly prominent in osteosarcoma, a tumor type that is highly dependent on microenvironmental regulation. These findings suggest that ICD is more likely involved in tumor–microenvironment co-evolution rather than acting solely as a unidirectional immune activation signal.

At the level of functional validation, this study further confirmed the biological role of the key gene NAP1L1 identified in the ICD risk model through *in vitro* experiments. Overexpression of NAP1L1 in MG-63 and U-2OS cells significantly promoted cell proliferation, migration, and invasion, consistent with its role as a risk factor. Beyond these oncogenic traits, we observed a significant correlation between NAP1L1 and key ICD effectors (e.g., CALR, HMGB1) in the clinical cohort. Mechanistically, NAP1L1 participates in chromatin remodeling (Andrews et al., 2010; Tanaka et al., 2019; Xiaohua et al., 2022). We hypothesize that NAP1L1-mediated chromatin compaction may dampen immunogenicity by transcriptionally repressing immune-related genes or hindering DAMP release. Thus, NAP1L1 likely drives a composite phenotype of aggressive growth and immune evasion, providing direct biological evidence supporting the high-risk features identified by our model.

Several limitations of this study should be acknowledged. First, although the functional role of NAP1L1 was validated *in vitro*, the complete molecular mechanisms underlying ICD regulation require further investigation in vivo models and at more refined pathway levels. Second, the single-cell analysis relies on the GSE152048 dataset, which includes 11 samples. While providing high-resolution insights into the TME, this limited sample size may not fully capture the complete spectrum of inter-patient variability or rare cellular phenotypes in osteosarcoma. Third, the drug sensitivity analyses are computational predictions based on *in vitro* pharmacogenomic data. These findings are hypothesis-generating and necessitate rigorous validation in vivo preclinical models or clinical trials to determine their therapeutic utility. Despite these limitations, this study integrates multi-level data to systematically construct an analytical framework for understanding ICD heterogeneity, prognostic value, and functional basis in osteosarcoma. These findings provide a solid foundation for the application of ICD in precision stratification and exploration of potential therapeutic targets.

## 5 Conclusion

This study systematically reveals the pronounced heterogeneity of ICD in osteosarcoma at single-cell resolution and demonstrates that ICD more closely represents an integrated transcriptional feature tightly associated with tumor cell states and microenvironmental interactions. An ICD related risk model constructed based on ICD-associated genes stably predicts patient prognosis across multiple independent cohorts and is closely linked to inflammation, metabolic reprogramming, cell–cell communication, and drug sensitivity differences. Together with *in vitro* functional validation of the key gene NAP1L1, this study provides systematic evidence supporting the biological significance of ICD in osteosarcoma and its potential application in precision stratification and exploration of therapeutic targets.

## Data Availability

The datasets presented in this study can be found in online repositories. The names of the repository/repositories and accession number(s) can be found in the article/supplementary material.
